# Recovery time and associated factors of severe acute malnutrition in children aged 0–59 months admitted to referral hospitals in Tanzania: an institution-based prospective cohort study

**DOI:** 10.3389/fnut.2025.1675898

**Published:** 2025-11-26

**Authors:** Anthony Samson, Elihuruma Eliufoo Stephano, Azaria Mugaya, Jackline Humphrey Mpembeni, Eusebi Kessy, Fredrick Ndunguru, Dina Mahamba, Arnold Gideon Lumbe, Mtoro J. Mtoro, Julius Ntwenya

**Affiliations:** 1 Department of Pediatrics, School of Medicine and Dentistry, The University of Dodoma, Dodoma, Tanzania; 2 Department of Clinical Nursing, School of Nursing and Public Health, The University of Dodoma, Dodoma, Tanzania; 3 Department of Public Health and Community Nursing, School of Nursing and Public Health, The University of Dodoma, Dodoma, Tanzania; 4 TILAM International, Dar es Salaam, Tanzania

**Keywords:** children, pediatrics, recovery time, severe acute malnutrition, under five

## Abstract

**Background:**

Malnutrition represents a pervasive global health challenge that severely impacts individuals of all ages. In Tanzania, severe acute malnutrition (SAM) is a critical health issue, contributing substantially to morbidity and mortality among children under the age of five. In light of the scarcity of local research on this topic, the present study aimed to evaluate the recovery time from SAM and identify its predictors among children aged 0 to 59 months.

**Methods:**

This hospital-based prospective cohort study enrolled 92 children aged 0–59 months with SAM who were admitted to two regional referral hospitals. Descriptive statistics were used to generate frequency distribution tables. A Cox proportional hazards regression model was used to identify factors associated with recovery time from SAM.

**Results:**

The recovery rate was 95.83%, with an average recovery time of 15 days. The predictors of recovery included children’s age, with those aged 25 to 55 months showing a tendency for quicker recovery (AHR = 1.1; 95%CI: 0.12–0.34; *p* = 0.001). Additionally, the children whose mothers or caregivers had university-level education were more likely to recover quickly (AHR = 1.56; 95%CI: 0.23–0.35; *p* = 0.003), indicating that the level of maternal or caregiver education positively influences the duration of recovery from SAM.

**Conclusion:**

The recovery rate and average recovery time observed in this study meet acceptable international standards. The factors associated with a higher likelihood of recovery included older child age and higher maternal or caregiver education levels. There is a greater need for targeted interventions at the community level to prevent SAM.

## Background

In children aged 6–59 months, severe acute malnutrition (SAM) is defined by a very low weight-for-height measurement of <-3SD. This condition is commonly characterized by bilateral lower limb edema and severe wasting or a mid-upper arm circumference (MUAC) of <115 mm (1). These children are at increased risk of morbidity and mortality due to compromised immunity (2). Globally, in 2025, 6.6% (42.8 million) of children aged 6–59 months experienced wasting and 1.9% (12.2 million) experienced severe wasting (3). These statistics show the persistent global burden of this condition over the past decade, since 2015 (3, 4). The 2022 Tanzania Demographic and Health Survey (TDHS) revealed a gradual decline in child malnutrition since 2015, with stunting decreasing from 34.4% to approximately 30%, wasting from 4.5% to approximately 3.1%, and underweight from 13.6% to approximately 12% (5). SAM is significantly influenced by social, political, and economic factors that directly affect food availability (6).

The World Health Organization (WHO) recommends hospital-based inpatient management for all SAM children who present with clinical signs of infection, metabolic disturbances, severe edema, anemia, dehydration, or lack of appetite (6). Recovery time from SAM is defined as the duration between admission and discharge. During this time, children must achieve clinical stability, remain free from complications, and attain an adequate nutritional status (characterized by an MUAC of ≥12.5 cm and a weight-for-height ratio of ≥85%) (7). Recovery time varies across different studies; however, a recent study reported a range of 8 to 47 days, while the SAM management guidelines recommend that recovery time should not exceed 4 weeks (6). The WHO developed SAM guidelines as part of its nutrition strategy to end hunger, achieve food security, and improve nutrition, in line with Sustainable Development Goals for 2025 (3). However, recovery outcomes vary significantly across different settings, with studies reporting recovery rates ranging from 25 to 95% in inpatient management and 50 to 93% in outpatient care (2, 8). Recovery time ranges from 8 to 43 days, depending on various patient- and facility-related factors (7, 8).

Multiple factors influence the recovery periods from SAM in children aged 0–59 months. These factors include patient characteristics such as age, weight gain velocity, the presence of edema, and comorbidities such as anemia, tuberculosis, diarrhea, and acute febrile illness (6). Treatment-related factors such as the provision of routine medications (such as vitamin A, folic acid, and antibiotics), specialized therapeutic interventions, early transition to Phase 2 treatment, and adequate nutritional rehabilitation also affect the recovery time from SAM (9). Recent studies from Ethiopia have demonstrated that children without comorbidities, those receiving appropriate therapeutic feeding, and those achieving adequate weight gain (more than 8 g/kg/day) experience significantly shorter recovery times (6); however, one study reported a median recovery time of 15 days (9).

Tanzania has reported improvements in malnutrition, reflecting the impact of targeted nutrition-specific and nutrition-sensitive interventions by the government and various partners that focus on maternal nutrition, child feeding, healthcare access, and disease prevention (10). The country has implemented child feeding programs such as the School Feeding Program, which follows the National School Feeding Guidelines (11). Additionally, other programs such as “*Pamoja Tuwalishe”* (12) and the School Milk Action Plan (13) contribute to these efforts. Other community interventions, such as Action Against Hunger, screen children for malnutrition and provide nutrition and health education (14) in accordance with the Infant and Young Child Feeding (IYCF) practices (15). Despite this progress, malnutrition remains a critical public health issue, affecting nearly one-third of children under the age of five, with persistent regional disparities and high absolute numbers due to population growth (16). A survey also highlighted poor infant and young child feeding practices, revealing that only 19% of children aged 6–23 months receive minimally diverse diets, which contributes to ongoing nutritional challenges (5).

Socioeconomic factors, including poverty, food insecurity, poor feeding practices, and limited access to healthcare services, further compound the burden. SAM management in the hospital usually involves several phases aimed at treatment and rehabilitation, often incorporating ready-to-use therapeutic foods (RUTFs) (17, 18). Despite the implementation of national nutrition programs and the adoption of the WHO guidelines for SAM management, limited research has been conducted to evaluate the recovery outcomes and identify the factors that influence treatment success in Tanzanian healthcare settings (19). This knowledge gap necessitates a comprehensive assessment of recovery time and the associated factors among children with SAM admitted to referral hospitals. This assessment will inform evidence-based improvements in treatment protocols and resource allocation strategies.

## Materials and methods

### Study design and setting

A prospective cohort study was conducted from 12 July to 22 August 2022 to assess the recovery time from SAM and identify its predictors among children aged 0–59 months. The study was conducted at Iringa and Dodoma Regional Referral Hospitals in Iringa and Dodoma regions, respectively. The hospitals provide inpatient services for the management of SAM. The Dodoma region is the capital city of Tanzania, and it has a population of 3,085,625, with female individuals accounting for 51% and male individuals for 49%. The Iringa region is located in the Southern Highlands zone of Tanzania, and it has a population of 1,192,728 (2022), with female individuals accounting for 52% and male individuals for 48% (20). The Iringa region has 349 health facilities, comprising 19 hospitals, 47 health centers, and 283 dispensaries. In total, 22 health facilities currently provide inpatient services for managing severe acute malnutrition in the region. The Dodoma region has 497 health facilities, comprising 26 hospitals, 69 health centers, and 402 dispensaries. In total, 17 health facilities provide inpatient services for managing severe acute malnutrition in the region.

### Study participants, sample size estimation, and sampling approach

The source population for this study was children aged 0 to 59 months, while the study population comprised children within the same age range who were diagnosed with severe acute malnutrition and were admitted to Iringa and Dodoma Regional Referral Hospitals during 2022. Each child admitted to the two hospitals served as a study unit. All children within this age range who presented with SAM were considered eligible for inclusion, provided that their parents or guardians gave informed consent to participate in the research. However, children were excluded from the study if their parents or guardians refused to cooperate or failed to provide consent, if they had incomplete or missing medical records that would compromise data quality, if they had documented secondary undernutrition resulting from underlying pathological disorders other than primary malnutrition, or if they presented with edema attributable to causes other than severe acute malnutrition. The sample size for this study was 96 participants. All cases of SAM recorded in the SAM register book and admitted during July 2022 were enrolled in the study.

### Data collection procedure

Maternal factors were assessed through a combination of a questionnaire and interviews with the mothers. The questions focused on the mothers’ feeding practices, economic status, level of education, and other sociodemographic factors affecting the recovery time. A standard semi-structured questionnaire created using Open Data Kit (ODK) was installed on mobile phones and translated into Swahili.

The assessment of child factors involved collecting data on the child’s age, sex, and anthropometric measurements at the time of admission. This information was obtained from the child’s Reproductive and Child Health (RCH) cards and hospital medical records. The presence of complications in the child, such as anemia, was clinically assessed or obtained from medical records if the condition had resolved by the time of the interview.

### Study variables

This study assessed the recovery time from SAM, with the dependent variable being the duration of recovery. This outcome variable was defined as the number of days from the participant’s admission to the hospital until their discharge. The recovery of the study participants was defined based on established national guidelines, which include achieving the specified weight-for-height percentage, the absence of edema, and the resolution of medical complications. All these data were extracted from hospital patient records.

The independent variables included the child’s age in completed months at the time of hospital admission; sex, which was categorized as male or female according to hospital records; child’s MUAC at the time of admission, which was measured in centimeters (cm) using a standardized MUAC tape; the child’s weight at admission, recorded in kilograms (kg) using a calibrated weighing scale; weight-for-height z-scores upon admission; the presence of complications or other infections; and any routine medications administered.

Other variables included the mother’s age in completed years at the time of the child’s admission, which was obtained through direct interviews using a structured questionnaire; marital status; education level, categorized as no formal education, primary education, secondary education, advanced education from advanced secondary school, or university education; occupation; and feeding practices. The independent variables were assessed through interviews using a questionnaire and by reviewing hospital records to obtain data on anthropometric measurements. During data collection, two clinical nurses were recruited as data collectors and one public health expert was appointed as a supervisor. All received 2 days of intensive training.

### Statistical methods

Data extracted from ODK software were processed and analyzed using STATA version 18. Information regarding age, weight, height, and edema was exported to Emergency Nutrition Assessment (ENA) for SMART software to calculate weight-for-height percentages and height-for-age Z scores at admission and discharge. Descriptive statistics were used to summarize the study’s sociodemographic characteristics and other relevant variables, which are presented as frequencies and proportions. The proportional hazards assumption was evaluated for the primary exposures and covariates using Schoenfeld residuals and log–log survival plots, and no substantial violations were detected. Therefore, a multivariable Cox proportional hazards regression model was used to determine the factors associated with recovery time from SAM. Statistical significance was set at a *p*-value of <0.05.

### Ethical approval and consent to participate

Ethical approval to conduct the research was obtained from the University of Dodoma Ethical Review Committee (MA.54/261/01/332). In addition, approval to conduct the research was granted by the Iringa Regional Authorities and Iringa Regional Hospital.

Informed consent was obtained from the research participants after thoroughly clarifying the study procedures. The participants were assured of anonymity and confidentiality. They were informed that participation in the study was voluntary, that the information collected would be kept confidential, and that it would be used solely for study purposes. The participants were assured that they were free to withdraw from the study at any time.

## Results

### Sociodemographic and baseline characteristics of children (0–59 months) at Dodoma and Iringa hospitals during admission, 2022 (*n* = 96)

Of the 96 children included in this study, the majority, 39 (40.63%), were aged 25–59 months. Both sexes were represented, with 56 (58.33%) male and 40 (41.67%) female individuals. The majority of the children, 68 (70.83%), had a breastfeeding history, while 28 (29.17%) had no breastfeeding history. The majority of the parents/caregivers, 40 (41.67%), had no formal education, 33 (34.38%) had primary education, 13 (13.54%) had secondary education, 6 (6.25%) had university education, and only 4 (4.17%) had advanced education. The majority of the mothers, 58 (60.42%), were engaged in peasant occupations (Table 1).

**Table 1:** Sociodemographic and baseline characteristics of the children (0–59 months) at Dodoma and Iringa hospitals during admission, 2022 (*n* = 96).

Variables	Category	*n*	%
Sex of the child	Male child	56	58.33
	Female child	40	41.67
Age of the child (months)	0–10	25	26.04
	12–24	32	33.33
	25–59	39	40.63
Breastfeeding history	Yes	68	70.83
	No	28	29.17
Maternal education	No	40	41.67
	Primary education	33	34.38
	Secondary education	13	13.54
	Advanced secondary education	4	4.17
	University education	6	6.25
Mother’s occupation	Self-employed as an entrepreneur	30	31.25
	Peasants	58	60.42
	Self-employed in business	2	2.08
	Government employee	6	6.25

### Routine medications

During the follow-up period for children with SAM, almost all participants (98.96%) received ampiclox and gentamicin as routine medications. In addition, 24 (25%) received albendazole or mebendazole, 11 (11.46%) received folic acid supplements, and 21 (21.87%) received vitamin A supplements.

### Medical comorbidity

Almost all children admitted to the study sites had at least one comorbidity. Over half of the children, 54 (57.24%), had diarrhea, and 41 (42.71%) reported the presence of illness with cough in the last 2 weeks. The majority of the children, 80 (78.6%), reported fever in the previous 2 weeks, and 31 (32.29%) were HIV-positive.

### Treatment outcomes

A total of 96 children with SAM were followed for periods ranging from 2 days to 59 days. The overall rate of recovery was 95.83%, while 2.08% of individuals defaulted and 2.08% died.

### Factors associated with recovery time from SAM

Various independent variables were analyzed using Cox proportional hazards regression in relation to the dependent variable. Two variables were significantly associated with recovery time from SAM. The children aged 25 to 55 months showed a tendency for quicker recovery (AHR = 1.1; 95%CI: 0.12–0.34; *p* = 0.001), which suggests that older children within this age group tend to recover more quickly from SAM. Additionally, the children whose mothers or caregivers had a university-level education were more likely to recover quickly (AHR = 1.56; 95%CI: 0.23–0.35; *p* = 0.003), indicating that the mother’s or caregiver’s level of education positively influences the duration of recovery from SAM (Table 2).

**Table 2:** Cox proportional hazards regression results show the factors associated with recovery from SAM among the children (0–59 months) at Dodoma and Iringa hospitals during admission, 2022 (*n* = 96).

Independent variables	Crude HR (95%CI)	Adjusted HR (95%CI)	*P*-value
Age of the child (months)			
0–11	Ref	Ref	**0.001**
11–24	1 (0.59, 1.69)	0.56 (0.25, 0.35)	
25–55	1.11 (0.67, 1.83)	1.1 (0.12, 0.34)	
Sex of the child			
Female child	Ref	Ref	0.450
Male child	1.03 (0.69, 1.54)	0.79 (0.68, 1.35)	
Education level of the mother/caregiver			
Advanced	Ref	Ref	**0.003**
None	0.75 (0.27, 2.11)	0.35 (0.11, 0.24)	
Primary	0.80 (0.28, 2.26)	0.33 (0.10, 0.18)	
Secondary	0.81 (0.27, 2.50)	0.37 (0.01, 0.11)	
University	0.75 (0.21, 2.67)	1.56 (0.23, 0.35)	
Fever			
Yes	Ref	Ref	0.504
No	0.82 (0.48, 1.42)	0.95 (0.34, 0.67)	
Cough			
Yes	Ref	Ref	0.854
No	1.04 (0.69, 1.55)	1.23 (0.57, 1.45)	
HIV status			
Positive	Ref	Ref	0.347
Negative	1.00 (0.65, 1.54)	1.97 (0.54, 1.00)	

HR, hazard ratio; Ref, reference group. Bolded means are statistically significant.

### Median recovery time

**Figure 1:**
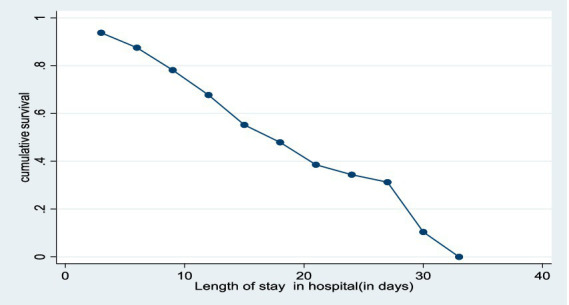
Length of hospitalization plotted against the cumulative survival of the children in the study.

The median nutritional recovery time was estimated to be 15 days [IQR: 95% CI (0.44–0.64)] (Figure 1).

## Discussion

This study aimed to assess recovery time and associated factors of SAM in children aged 0–59 months admitted to Dodoma and Iringa referral hospitals in Tanzania using an institution-based prospective cohort design. This study estimated the overall recovery rate from SAM to be 95.83%, which exceeds the accepted international Sphere standard (>75%) (21). Additionally, this rate is higher than the recovery rate reported in a study conducted across 12 hospitals in Ethiopia (22). However, another study reported a lower recovery rate compared to our findings (23). This difference might be attributed to fewer comorbidities, improved effectiveness of nutritional programs, and a lower patient load, all of which lead to improved care and favorable outcomes. Tanzania is implementing the Integrated Management of Acute Malnutrition, which combines inpatient treatment with outpatient care, facilitating faster recovery (17).

Moreover, the average recovery time was 15 days, which falls within the accepted international Sphere standard (<28 days) (24, 25). The majority of the children recovered within the first month, which is consistent with another study reporting an average recovery time within the recommended period of fewer than 28 days (26). This similarity might be due to the similarities between the studies and the involved population. However, contrary to our findings, a survey in northeastern Ethiopia showed an average recovery time of 11 days, indicating a faster recovery compared to our findings (27). This difference is due to variations in severity at admission, improved therapeutic programs, availability of skilled staff and resources, and a lower patient load, all of which lead to improved patient care.

The study showed a significant association between age and recovery from SAM, with older children recovering more quickly compared to younger children. The results underscore how age stands out as an essential explanatory and predictive factor for the recovery trajectory in children with SAM, consistent with findings from a study conducted in Southern Ethiopia (28). Nutritional strategies are crucial for optimizing recovery outcomes in older children (27). This group has unique needs and responses to treatment, highlighting the importance of designing interventions that address these needs (28). Further research should explore the factors contributing to age-related differences in recovery time, enabling more precise and effective design of nutrition interventions.

Our findings also indicated that there exists a correlation between the education level of mothers or caregivers and the recovery time of children with SAM, where those whose mothers or caregivers have a higher educational background tend to recover more quickly. Similar findings were noted in a study conducted in the Amhara region, Ethiopia (29). Another study reported that low maternal education was associated with SAM (30), whereas an additional study showed that maternal education was associated with improvements in SAM (31), which is in line with our findings. The results underline the potential impact of maternal education on nutritional outcomes among children. Therefore, our findings indicate the necessity of implementing targeted educational and support interventions to further improve recovery rates and enhance the overall well-being of children experiencing SAM.

### Limitations of the study

The study used some secondary data with incomplete records for certain variables. The researcher also failed to explore factors such as household income and distance from the facility.

## Conclusion

This study provides important insights into the recovery time and factors associated with the recovery of children with SAM admitted to referral hospitals. The overall recovery rate from SAM at 95.83% was higher than international thresholds, suggesting that nutritional programs has increased patient care. The majority of the children recovered within the first month, with an average recovery duration of 15 days, which aligns well with accepted treatment standards. Age emerged as a significant factor, with older children displaying a tendency toward more rapid recovery. This finding emphasizes the need to develop tailored nutritional strategies for different age groups. Maternal or caregiver education also proved to be a key factor, as the children whose mothers or caregivers had higher levels of education tended to recover more quickly. In addition, the absence of comorbidities, especially HIV, was associated with a shorter recovery time. The fact that there were mixed findings underscores the multifaceted nature of factors influencing recovery from SAM. There is a strong need for targeted interventions based on age, maternal education, and comorbidity status to achieve optimal outcomes in the management of severe acute malnutrition among children.

## Data Availability

The raw data supporting the conclusions of this article will be made available by the authors, without undue reservation.
